# Synergistic Regulation of Tumor Immunity by Integrins and Lectins: From Molecular Mechanisms to Dual-Targeted Therapy

**DOI:** 10.3390/ijms27188245

**Published:** 2026-09-16

**Authors:** Rongyang Liu, Chen Liu, Xiaotong Guo, Peiyan Li, Yewei Niu, Jiawei Zhao, Jingyi Zhang, Xiaolin Su, Jian Chen, Jiamin Jin, Jinfeng Yang

**Affiliations:** 1 Department of Immunology, Guilin Medical University, Guilin 541199, Chinachjian80726@163.com (J.C.); 2 Key Laboratory of Tumor Immunology and Microenvironmental Regulation, Guilin Medical University, Guilin 541199, China; 3 College of Pharmacy, Heilongjiang University of Chinese Medicine, Harbin 150040, China

**Keywords:** integrin, lectin, tumor immune microenvironment, synergistic regulation, targeted immunotherapy, combined therapy

## Abstract

The high invasiveness and metastatic potential of malignant tumors represent major obstacles to successful clinical treatment and are closely associated with poor patient prognosis. These processes rely heavily on the dynamic remodeling of the tumor microenvironment. Within this milieu, integrins—a family of transmembrane receptors mediating cell–matrix and cell–cell interactions—along with lectins capable of recognizing specific carbohydrate structures, form a complex and coordinated regulatory network that collectively drives tumor progression. This review systematically elucidates the key mechanisms by which this network regulates the malignant phenotype of tumor cells, mediates microenvironmental interactions, induces therapy resistance, and reshapes the immunosuppressive tumor microenvironment. Drug development strategies targeting this network have evolved from single-target inhibition toward intervention in coordinated signaling pathways. However, clinical translation remains challenged by tumor heterogeneity, complex resistance mechanisms, and off-target toxicity. Therefore, future efforts aimed at deepening our understanding of their regulatory roles within the tumor immune microenvironment, along with optimizing immune-based combination therapies, will provide valuable insights for both basic research and clinical translation in this field.

## 1. Introduction

### 1.1. Research Background and Significance

The high invasiveness and metastatic nature of malignant tumors are the key reasons for clinical treatment failure and poor patient prognosis. The tumor microenvironment (TME), which is a complex ecosystem composed of various components such as tumor cells, immune cells, vascular endothelial cells, cancer-associated fibroblasts (CAFs), and extracellular matrix (ECM), undergoes dynamic changes that are closely related to tumor progression [1]. Integrins, as transmembrane receptors that connect the intracellular skeleton with the extracellular environment, can sense and transduce microenvironmental signals, thereby regulating multiple downstream signaling pathways related to tumor growth, survival, and invasion and metastasis [2]. At the same time, proteins encoded by lectin genes specifically recognize and bind to carbohydrate structures on cell surfaces or extracellular matrices through their sugar recognition domains, participating in key processes such as cell communication and immune regulation [3]. Together, they constitute an important collaborative regulatory network in the TME [4]. Recent studies have shown that members of the integrin family and various lectins are abnormally co-expressed in multiple malignant tumors and are significantly associated with tumor stage, metastatic potential, and poor patient prognosis, suggesting their potential as combinatorial biomarkers or targets [5,6]. However, existing single-target treatment strategies in clinical applications often have limited response rates and are prone to drug resistance, thus urgently requiring in-depth analysis of the collaborative regulatory mechanisms of integrins and lectin genes in the TME, in order to provide a solid theoretical basis for the development of more efficient and durable combined treatment strategies.

### 1.2. Overview of Integrins and Lectin Families

Integrins are formed by a heterodimer of two subunits, α and β, and 8β subunits have been identified, forming 24 different integrin heterodimers. Different subunit combinations give integrins different ligand-binding specificities, with ligands mainly including ECM components and cell surface molecules [7]. The functional regulation of integrins mainly relies on their activation state transition, that is, from a low-affinity “resting state” to a high-affinity “activated state”, which is regulated by both intracellular signals and extracellular ligand binding-mediated extracellular signals [8]. The head domain of talin and kindlin and intracellular adaptor proteins play key roles in the activation of integrins.

The activation process of talin is also finely regulated by its tail domain in the cytoplasm, which binds to the cytoplasmic tail of the β subunit of integrins, initiating the inward signal transduction and promoting the connection of integrins with the actin cytoskeleton. For example, α-actinin can competitively bind to integrins and induce kinking in the transmembrane region of the β subunit, thereby impairing integrin signal transduction [9]. Additionally, filamin proteins can directly interfere with the activation mediated by talin [10]. The latest single-molecule studies have further revealed that ligand binding can trigger the coordinated extension and opening of the legs and the opening of the head of integrins within milliseconds, while talin binding can stabilize integrins, but is not sufficient to induce their complete extension and opening alone, emphasizing the necessity of the transmission of force through the “ligand-integrin-talin-microfilament” complex for complete activation [11].

Lectin gene family members are widely distributed in animals, plants, and microorganisms. According to the structural characteristics of their carbohydrate-recognition domains (CRDs), they can be divided into multiple subfamilies such as C-type, S-type (galectins), and P-type [12]. The galectin family is one of the most studied subfamilies in tumor research. The activity of classical C-type lectins depends on calcium ions and can recognize pathogen- or tumor-associated glycans, playing a key role in immune recognition [13]. Galectins family are represented by the family of galactoside-binding lectins (galectins) are β-galactoside-binding lectins, among which galectin-3 is a research hotspot; its Trp181 and Glu184 residues are crucial for high-affinity binding to β-galactosides [14]. P-type lectins mainly mediate the targeted transport of lysosomal enzymes [15]. Galectins specifically bind to glycan structures containing galactose, mannose and other residues through their conserved CRDs. Their functional regulation depends largely on oligomerization state and intracellular versus extracellular localization; oligomerized galectins enhance glycan affinity through multivalent binding, thereby efficiently regulating downstream signaling [16].

Under physiological conditions, integrins and selectins/galectins synergistically mediate cell adhesion and signal transduction and jointly participate in processes such as lymphocyte homing. Selectins mediate the initial tethering and rolling, whereas integrins mediate firm adhesion and transmigration [13]. Under pathological conditions, dysfunction or aberrant synergy of both is closely related to various diseases. In tumors, they jointly drive malignant progression: integrin signaling and galectin-3 synergistically promote tumor cell invasion, metastasis and drug resistance [4], and a cancer cell-specific type I collagen homotrimer in pancreatic cancer signals through α3β1 integrin to suppress CXCL16 and reduce CD8^+^ T cell infiltration [17]. In autoimmune diseases, aberrant lymphocyte migration mediated by integrin α4β1/α4β7 is a core process, and monoclonal antibodies targeting these integrins have become effective therapies [18]; Tivation mediated by integrin αvβ6 and fibroblast activation driven by galectin-1 functionally cooperate to drive tissue fibrosis [19,20]. It is worth noting that this synergy is not limited to oncology. In infectious diseases, pathogens hijack host integrins as invasion receptors, while the C-type lectin receptor DC-SIGN (Dendritic Cell-Specific Intercellular Adhesion Molecule-3-Grabbing Non-integrin; CD209) recognizes glycans on pathogen surfaces and mediates immune recognition or pathogen escape [21]. In tissue damage and repair, integrin-mediated cell migration and galectin-regulated inflammatory responses jointly determine repair outcome.

### 1.3. Review Objectives and Scope

This article aims to provide a narrative review of the core biological characteristics of integrins and lectins, with a focus on their synergistic regulatory functions in the tumor microenvironment, including the regulation of tumor cell’s own malignant phenotype, the mediation of interactions between tumor microenvironment components, and the influence and molecular mechanisms on key processes of tumor progression; summarize the current types of targeted drug development based on integrin and lectin genes, clinical application progress, and challenges; deeply explore the regulatory mechanisms of both lectins and integrins in the tumor immune microenvironment and optimization strategies for immune combination therapy, providing comprehensive references for basic research and clinical translation in this field.

## 2. Regulatory Functions of Integrins and Lectins in the Tumor Microenvironment

### 2.1. Regulatory Functions of Lectins in the Tumor Microenvironment

Lectins, through their sugar recognition domains, specifically bind to abnormal glycosylation structures on the surface of tumor cells, exerting multi-level regulatory functions in the tumor microenvironment. Members of the C-type lectin receptor family, such as dendritic cell immunoreceptor (DCIR), macrophage galactose-type lectin (MGL), dendritic cell-associated C-type lectin-1 (Dectin-1), and C-type lectin domain family 4 member E (CLEC4E), can directly regulate the proliferation, migration, and invasion of tumor cells, and participate in tumor progression by influencing the function of immune cells. For example, Dectin-1 can promote colorectal cancer development through downstream signaling pathways, while CLEC4E is highly expressed in tumor-associated macrophages and drives immunosuppressive [22]. Galectin-1, a member of the galectin family, can establish an immunosuppressive microenvironment by inducing T cell apoptosis and promoting the recruitment of regulatory T cells (Tregs) and myeloid-derived suppressor cells (MDSCs) [23]. Galectin-3 secreted by triple-negative breast cancer (TNBC) cells can inhibit anti-tumor immunity by inducing the expansion of Tregs and causing mitochondrial dysfunction and exhaustion of CD8+ T cells, thereby inhibiting anti-tumor immunity and promoting tumor progression and metastasis [24]. In hepatocellular carcinoma (HCC), Galectin-4 stabilizes LDHA to enhance glycolysis, thereby activating the HIF-1α/CXCL6 axis, promoting the accumulation of PD-L1+ tumor-associated neutrophils (TANs) and damaging the function of CD8+ T cells, thereby driving resistance to atezolizumab combined with bevacizumab treatment [25]. In colorectal cancer (CRC), the highly sialylated glycan chains on the surface of tumor cells bind to Siglec-15 receptors, inhibiting T cell function, thereby promoting immune escape and tumor progression. These lectins, through a complex glycosylation recognition network, jointly shape the tumor immune microenvironment and, in some cases, directly mediate resistance to specific therapies (as exemplified by Galectin-4 in HCC driving resistance to atezolizumab/bevacizumab). This dual role provides a potential new strategy for combined targeted therapy.

### 2.2. Regulatory Role of Integrins in the Tumor Microenvironment

Integrins, as core transmembrane receptors connecting cells and ECM, not only mediate physical adhesion of tumor cells, but also profoundly affect their malignant phenotypes such as proliferation, migration, invasion, survival, and immune escape through a complex bidirectional signaling network. They can precisely sense changes in mechanical properties such as stiffness, composition, and fluid shear force of the ECM, and convert physical signals into biochemical signals through the integrin-adhesion plaque complex, activating downstream pathways including focal adhesion kinase (FAK)/Src, Ras homolog (Rho)/Rho-associated coiled-coil containing protein kinase (ROCK), and Hippo/Yes-associated protein (YAP)/transcriptional coactivator with PDZ-binding motif (TAZ) [26]. For example, in rigid matrix, integrins enhance the connection with the actin cytoskeleton, drive the activation of adhesion kinase and Rho GTPases, disrupt epithelial cell polarity, and induce epithelial–mesenchymal transition (EMT), thereby endowing tumor cells with stronger migration and invasion capabilities [27]; In addition, integrins also widely regulate the behavior of other components in the TME: they mediate the interactions between tumor cells and CAFs, immune cells, and endothelial cells, promoting the secretion of immunosuppressive cytokines and chemokines, recruiting and polarizing Tumor-Associated Macrophages (TAMs) and Tregs, thereby shaping an immunosuppressive microenvironment [2].

**Figure 1:**
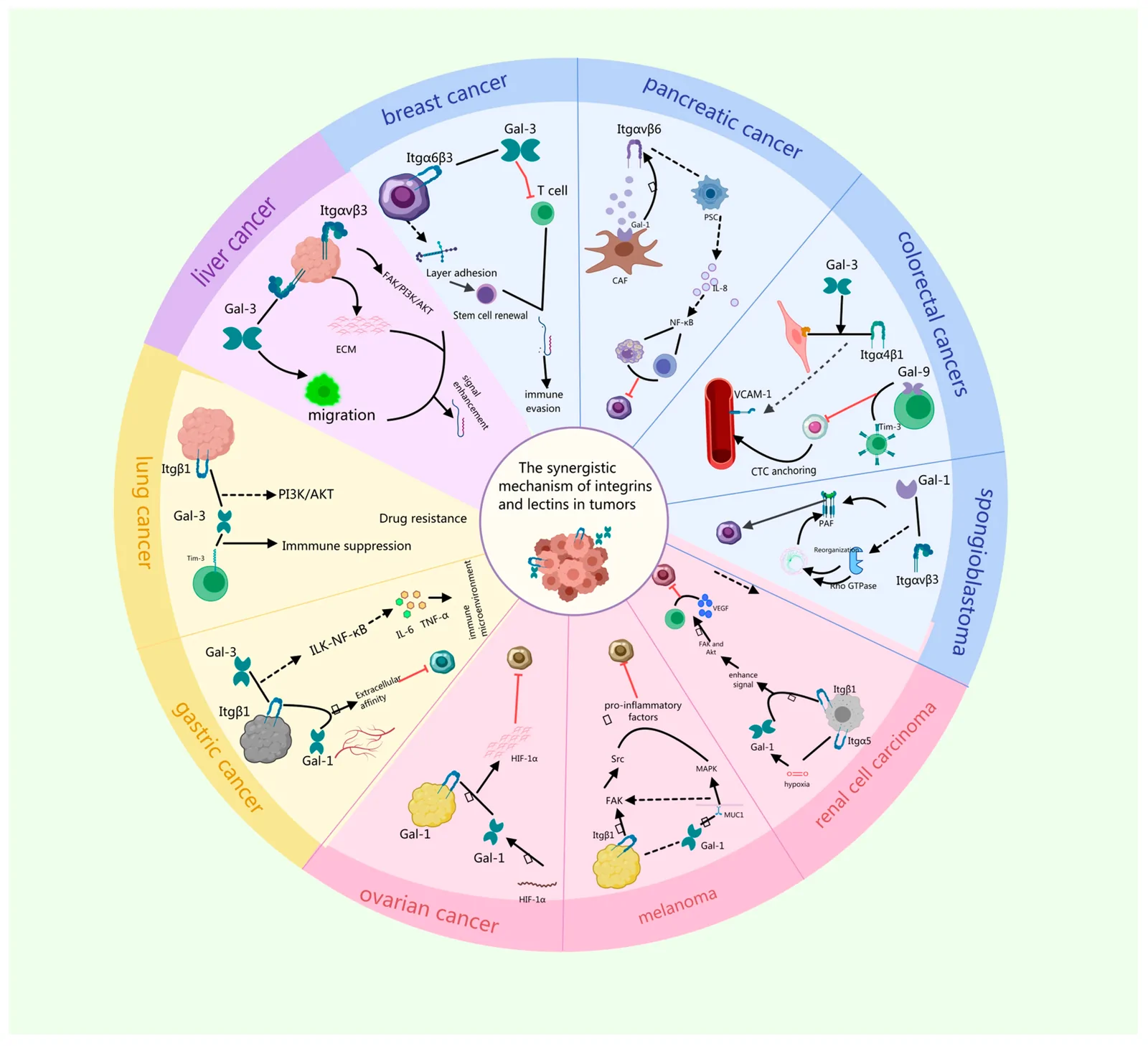
Overview of the mechanisms by which integrins and lectins regulate the tumor microenvironment. (Created with MedPeer, medpeer.cn) Integrins and lectins exert their effects on the progression and metastasis of tumors through a variety of mechanisms. Direct molecular interactions (e.g., lectin binding to integrin glycans) are distinguished from indirect pathway convergence (e.g., parallel activation of downstream survival signals). “Solid arrows indicate direct molecular interactions (e.g., lectin binding to integrin glycans); dashed arrows indicate indirect pathway convergence or downstream effects; curved arrows represent positive feedback loops.”.

Current studies have revealed that integrin α3β1 can directly inhibit the phagocytic function of macrophages by binding to Siglec-10 on their surface, promoting immune escape in pancreatic cancer [28]. The integrin β1/FAK/YAP axis can be activated by highly hard extracellular matrices. By upregulating the deubiquitinating enzyme ubiquitin-specific protease 8 (USP8), it stabilizes immune checkpoint molecules such as PD-L1, helping prostate cancer evade T cell killing [29]. Meanwhile, integrins αvβ3 and αvβ5 play key roles in tumor angiogenesis, activating the vascular endothelial growth factor (VEGF) signaling pathway in a ligand-independent manner, promoting endothelial cell migration and lumen formation [30]. These integrins, through multiple mechanisms such as biochemical signaling, directly driving the malignant behavior of tumor cells, and reshaping the immune and vascular microenvironment, constitute the core regulatory network of tumor progression, making them highly potential multi-dimensional therapeutic targets (Figure 1).

### 2.3. Synergistic Regulatory Functions of Integrins and Lectins in the Tumor Microenvironment

For the purpose of this review, we define “synergy” as an interaction between integrins and lectins that meets at least one of the following operational criteria: (1) Direct physical interaction, such as lectin binding to integrin glycans to alter its conformation or clustering; (2) Positive feedback regulation, where activation of one component enhances the expression or function of the other, creating an amplification loop; or (3) Convergent signaling, where both molecules act on a common downstream pathway to produce an effect greater than the sum of their individual contributions. A prime example is the “integrin- lectin- glycosylation” axis, which cross-talks with immune checkpoint pathways such as PD-1/PD-L1, thereby synergistically promoting immune escape.

#### 2.3.1. Regulation of Tumor Cell Malignant Phenotype

Integrins and lectins have significant coordinated functions in regulating the malignant phenotype of tumor cells. Integrins, as the core adhesion receptors, mediate cell–cell and cell–ECM interactions and activate downstream signaling pathways such as FAK/Src, PI3K/AKT, and Rho/ROCK, directly driving cytoskeleton remodeling [31], thereby affecting the migration, invasion, and survival of tumor cells. Lectins, through their sugar recognition domains, specifically recognize and bind to glycan structures on integrins and other membrane proteins, or directly interact with integrins, thereby enhancing and amplifying pro-tumor signals. For example, in liver cancer, the integrin αvβ3 binds to fibronectin in the ECM, activating pro-migration signals [32], while galectin-3 can bind to the glycan chain of this integrin and further stabilize the complex and enhance the activation of downstream ERK signaling, functionally cooperating to promote the movement and invasion of tumor cells [33]. Additionally, aberrantly activated integrin signals can promote tumor cell proliferation and inhibit apoptosis, while lectin family members such as galectin-9 can enhance the cell’s anti-apoptotic ability by regulating the expression of apoptosis-related proteins [34]. In the maintenance of stemness, integrin α6 and galectin-3 are often co-expressed in breast cancer stem cells (BCSCs), and they may jointly contribute to stem cell-related pathways such as Wingless/Int (Wnt)/β-catenin through cross-talk, thereby potentially enhancing the self-renewal ability and drug resistance of tumor cells [35].

#### 2.3.2. Mediation of Interactions Between Components of the Tumor Microenvironment

Integrins and lectins jointly mediate complex interactions among different components of the TME. In the interaction with CAFs, the integrins expressed by tumor cells bind to ECM components such as fibronectin and collagen secreted by CAFs, not only providing physical anchoring but also promoting the activation and proliferation of CAFs through bidirectional signaling [36]. At the same time, galectin-1 secreted by tumor cells can recognize and bind to the highly glycosylated receptors on the surface of CAFs, such as CD146, further activating the TGF-β/Smad signaling pathways in CAFs [37], enhancing their activation and pro-tumor factor secretion, and activated CAFs express integrin α11β1 to accelerate matrix remodeling and secrete galectin-3, which, together with the integrin signal, promotes collagen cross-linking and tissue fibrosis, creating physical channels for tumor invasion [38].

During tumor angiogenesis, integrins and lectins play a core coordinated regulatory role. Tumor cells secrete cytokines such as VEGF to induce high expression of integrin αvβ3 and αvβ5 in endothelial cells, promoting their proliferation, migration, and tube formation [39]. Galectin-1 expressed by tumor cells and endothelial cells themselves can bind to the N-linked glycan chain of integrin α5β1 on the surface of endothelial cells, allosterically regulating the conformation of integrins, enhancing their affinity for ligands, and stabilizing the adhesion plaque complex and promoting endothelial cell activation [40]; during the hematogenous metastasis stage, integrins and lectins on the surface of circulating tumor cells jointly mediate specific adhesion to vascular endothelial cells, laying the foundation for tumor extravasation.

In the process of immune regulation and immune escape, integrins and lectins coordinately shape an immunosuppressive TME through multiple mechanisms. The integrins on the surface of tumor cells regulate the interaction between the cells and the ECM, thereby influencing the chemotaxis and infiltration of immune cells [41]. Integrins can also participate in the induction of immunosuppressive cytokine secretion through mediated signals [42]. Tumor cell-derived lectins, such as galectin-3, can interfere with the activation signals of T cells by interacting with specific glycosylated proteins on the surface of T cells, inhibiting their proliferation and promoting their apoptosis [43]. In terms of antigen-presenting cells, certain integrin signals have been confirmed to inhibit the maturation process of dendritic cells (DCs) and weaken their antigen-presenting ability. Additionally, integrins and lectins functionally cooperate with each other to promote the recruitment, retention, and functional activation of immunosuppressive cells such as Tregs and myeloid-derived suppressor cells (MDSCs) at the tumor site [44]. For example, galectin-9 on the surface of tumor cells can mediate the directional aggregation of regulatory T cells by binding to the immune checkpoint receptor T cell immunoglobulin and mucin-domain containing-3 (Tim-3) on T cells, while the adhesion and migration signals mediated by integrins help these cells stabilize their residence in the tumor tissue and exert immunosuppressive functions [45].

#### 2.3.3. Regulation of Tumor Resistance

The abnormal coordinated activation of integrin and lectin signaling pathways is an important mechanism leading to tumor resistance to chemotherapy, targeted therapy, and immunotherapy. In chemotherapy resistance, integrins activate downstream signaling pathways such as FAK/PI3K/AKT or integrin-linked kinase (ILK) to enhance the anti-apoptotic ability of tumor cells [46]. Galectin-3 can further enhance the activity of these pathways and upregulate the expression of multidrug resistance proteins, jointly reducing the cell death induced by chemotherapy drugs [47,48]. In targeted therapy resistance, for example, for anti-epidermal growth factor receptor (EGFR) therapy, tumor cells can activate the bypass signaling pathway through the integrinβ1/Src signal, bypassing EGFR inhibition [49]. Additionally, the abnormal glycosylation modification of Galectin-9 mediated N-acetylglucosaminyltransferase IVa (GnT-IVa, encoded by MGAT4A; official full name: alpha-1,3-mannosyl-glycoprotein 4-beta-N-acetylglucosaminyltransferase A) can further enhance integrin signal transduction, representing an upstream–downstream regulatory mechanism that contributes to drug resistance [50]. In immune therapy resistance, both of them coordinately function to maintain a highly immunosuppressive microenvironment, thereby weakening the efficacy of immune checkpoint inhibitors (ICIs) [51]. For example, in the treatment with integrin inhibitors such as cilengitide, tumor cells with high expression of galectin-3 can continuously activate the residual integrin-related survival signals [47], leading to resistance. More directly, galectin-3 acts as a glycan scaffold for PD-1/PD-L1 and sterically blocks pembrolizumab/atezolizumab binding, an effect reversed by the galectin-3 inhibitor GB1211 [51]. MGAT4A/Galectin-9 modification of the Glucose transporter 1 (GLUT1) glycan structure enhances glucose uptake and cooperates with integrin signaling to promote tumor proliferation and invasion, participating in the regulation of drug resistance mechanisms [52]. Additionally, galectin-9 can enhance the glucose uptake ability of tumor cells by modifying the glycan structure of glucose transporter 1 (GLUT1), and this metabolic reprogramming can operate in parallel with integrin-driven PI3K/AKT/mechanistic target of rapamycin (mTOR) signaling to promote tumor progression. Together, they participate in the formation of multiple treatment resistance mechanisms [47]. In summary, integrin and lectin-mediated resistance is not a static outcome but a dynamic adaptation process. Its core can be summarized as chemotherapy, targeted therapy, and immunotherapy may trigger the reprogramming of integrin signals and the upregulation of lectin expression, which, through enhancing cell–matrix adhesion, activating downstream survival pathways, reshaping the immune microenvironment, and reprogramming cellular metabolism, collectively construct a multi-level, complementary defense network. Importantly, these represent distinct mechanisms—upstream–downstream regulation and functional complementarity—rather than a single synergistic process. Therefore, simultaneously targeting multiple pillars is a key strategy to overcome resistance.

## 3. Integrin and Lectin in Specific Cancer Types

### 3.1. Hepatocellular Carcinoma

In HCC, members such as integrin β1 and β4 are abnormally highly expressed, and are significantly associated with local invasion of the tumor, low differentiation degree, and poor prognosis [53]. Integrin β4 activates the AKT/HK2 signaling pathway to drive glycolysis, promotes lactate secretion, and subsequently recruits and activates cancer-associated fibroblasts, shaping the pre-metastatic microenvironment [54]. Meanwhile, galectin-3 binds to integrin β1, activates the ILK-NF-κB signaling axis, promotes IL-8 secretion, and maintains the tumor microenvironment homeostasis [55]. Both act synergistically through the “integrin-lectin-glycosylation” circuit to enhance the activity of downstream pathways such as ERK and FAK, jointly driving the migration, invasion, and drug resistance of hepatocellular carcinoma cells [56]. Therapeutically, the integrin αvβ3 inhibitor cilengitide combined with the extract of prekine can synergistically inhibit the adhesion of hepatocellular carcinoma cells [57].

### 3.2. Breast Cancer

Integrin β1 in TNBC interacts with CD47 through lipid raft enrichment and activates the Integrinβ1/FAK signaling, promoting metastasis. Kindlin-2 acts as a linker protein, stabilizing the complex of β1-integrin and TGF-β1 receptor, driving downstream oncogenic pathways [58]. Integrin α6β1, as a key marker of breast cancer stem cells, mediates adhesion to laminin and maintains stem cell properties, self-renewal, and promotes metastasis. Lectin families such as galectin-3 receptors are used to design targeted peptides, enhancing tumor-specific recognition. Integrins and lectins jointly regulate the tumor microenvironment [24]. Integrin signaling promotes the activation of CAFs and matrix remodeling, while Galectin-3 enhances integrin-mediated adhesion and signal transduction by binding to membrane protein glycans, jointly maintaining an immunosuppressive state [59].

Therapeutic strategies include: peptide drugs targeting integrins; immunotherapy combined with other treatments, such as PD-1/PD-L1 inhibitors combined with anti-angiogenic drugs or antibody–drug conjugates (ADCs) drugs, which have shown survival advantages in PD-L1-positive triple-negative breast cancer (TNBC).

### 3.3. Pancreatic Cancer

Integrins αvβ6, αvβ3, β4, etc., are highly expressed in pancreatic cancer and drive proliferation, invasion, and immune escape by activating pathways such as FAK/Src, PI3K/AKT, TGF-β/Smad. Integrin αvβ6: efficiently activates TGF-β, is a core molecule driving matrix fibrosis, epithelial–mesenchymal transition (EMT), and immunosuppression. A peptide–drug conjugate (PDC), SG3299, developed on the basis of the A20FMDV2 peptide derived from foot-and-mouth disease virus (FMDV), can selectively kill αvβ6-expressing pancreatic cancer cells [60]. Tyrosylprotein sulfotransferase 2 (TPST2) mediates the sulfation of integrin β4 to stabilize its expression and promote pancreatic cancer progression [61]. Galectin-1 activates pancreatic stellate cells (PSCs) through integrin β1 to produce interleukin-8 (IL-8), enhances nuclear factor kappa B (NF-κB) signaling, and promotes tumor–stroma interactions [62]. Both coordinately function to shape a highly immunosuppressive microenvironment: Integrin signaling inhibits T cell infiltration, while Galectin-3 directly induces T cell apoptosis; simultaneously, Integrin αvβ8 activates the TGF-β signaling and upregulates PD-L1 expression, weakening the immune treatment response [63].

Treatment progress includes the following: Integrin αvβ6 is an important therapeutic target. Peptide-drug conjugates based on its ligand and radioisotope therapy have shown potential for selective killing of tumor cells in preclinical and early clinical studies. Other subtypes, such as Integrin α3, have been proven to be related to tumor heterogeneity and resistance. Siglecs, members of the lectin family, act as emerging sugar immune checkpoints, and the mediated tumor–immune cell interactions are a key mechanism for regulating the immunosuppressive microenvironment. Inhibitory antibodies related to this have shown efficacy in bone metastasis models.

### 3.4. Colorectal Cancer

In colorectal cancer, integrins and lectin families coordinately reshape the immune microenvironment, becoming key drivers of tumor immune escape. C-type lectin receptors (CLRs) such as dendritic cell-associated C-type lectin-1 (Dectin-1) and dendritic cell immunoreceptor (DCIR) promote the synthesis of prostaglandin E2 (PGE_2_) by myeloid-derived suppressor cells (MDSCs) and inhibit the expression of interleukin-22 binding protein (IL-22BP, also known as IL22RA2), thereby establishing an immunosuppressive state [64]. Galectin-3 enhances cell adhesion and proliferation; galectin-9 acts as an immune regulatory molecule that can induce T cell apoptosis or exhaustion; galectin-1 induces CD8^+^CD122^+^PD-1^+^ regulatory T cells with strong immunosuppressive function, further inhibiting the anti-tumor immune response, and its high expression is significantly associated with poor patient prognosis [65]. Integrin α5β1 and αvβ3 not only mediate the adhesion of tumor cells to the extracellular matrix to promote metastasis, but also cross-talk with the lectin pathway by activating FAK/TGF-β and other signaling axes to upregulate the expression of immune checkpoint molecules such as PD-L1, thereby helping tumor cells evade T cell attack [66].

Treatment progress: Combined therapy is the core breakthrough: ① Cetuximab combined with PD-1/PD-L1 inhibitors and irinotecan can overcome EGFR signal-mediated resistance [67]; ② anti-angiogenic drugs (bevacizumab) combined with immunotherapy show survival benefits in patients with RAS mutations/MSS type [68]; ③ new integrin inhibitors (GLPG-0187) can reverse the immunosuppressive microenvironment by downregulating the TGF-β/PD-L1 axis, and produce a synergistic effect with immune checkpoint blockers [69].

### 3.5. Glioblastoma

Integrin αvβ3 and αvβ5 are crucial in the angiogenesis and maintenance of tumor stem cells in glioblastoma (GBM) [70]. They collaborate with vascular endothelial growth factor receptor 2 (VEGFR2, also known as kinase insert domain receptor [KDR]/Flk-1) to promote endothelial cell proliferation and vascular co-option; galectin-1 (Gal-1) is the core driver of immunosuppression in GBM, secreted by tumor cells and tumor-associated macrophages, and induces T cell apoptosis and promotes regulatory T cell infiltration [71]. The collaborative network is exemplified by the following regulatory axis: integrin-mediated cell adhesion activates hypoxia-inducible factor 1-alpha (HIF-1α, encoded by HIF1A), leading to upregulated expression of Gal-1. In turn, Gal-1 further stabilizes integrin signaling and enhances the self-renewal capacity of cancer stem cells via activation of the H-Ras (Harvey rat sarcoma viral oncogene homolog, HRAS)/PI3K pathway, as evidenced by studies on specific cancer model [72]. Due to the blood–brain barrier limitation, the treatment strategies focus on local delivery and new targets: the clinical trial of the integrin αvβ3/αvβ5 inhibitor cilengitide combined with radiotherapy and chemotherapy failed to significantly improve overall survival, but it highlighted the necessity of targeting the tumor microenvironment [73].

### 3.6. Lung Cancer

Integrins αvβ3, α5β1 and α6β4 play a crucial role in the invasion, metastasis, and resistance to epidermal growth factor receptor tyrosine kinase inhibitors (EGFR-TKIs) in non-small cell lung cancer (NSCLC) [74]. The integrin β1 signal is one of the key bypass mechanisms for EGFR-TKI acquired resistance [49]. The integrin αvβ3 forms a complex with EGFR, activating downstream FAK/Src and PI3K/AKT signals, bypassing EGFR inhibition, and leading to resistance to drugs such as osimertinib [75,76]. Galectin-1 is highly expressed in CAFs and tumor cells, inducing T cell apoptosis and promoting regulatory T cell function, thereby shaping an immunosuppressive microenvironment [77]. The two coordinately function as follows: under EGFR-TKI pressure, the unliganded integrin αvβ3 recruits KRAS via galectin-3, activating the RalB–TBK1–NF-κB axis to sustain survival—a feed-forward loop that operates independently of integrin ligation [78]. In terms of treatment, peptidomimetic drugs targeting integrin αvβ3 combined with EGFR-TKIs show synergistic effects in preclinical models [79]; galectin-1 inhibition combined with PD-1/PD-L1 blockade reverses T cell exclusion and enhances the efficacy of immunotherapy in preclinical studies [80].

### 3.7. Gastric Cancer

Integrin αvβ6 and β1 are overexpressed in gastric cancer and are closely related to peritoneal metastasis and poor prognosis. Integrin αvβ6 activates local adhesion kinases and TGF-β signaling, driving epithelial–mesenchymal transition and invasion [81]; the family member of the lectin family, Galectin-3, binds to integrin β1 and jointly activates the ILK-NF-κB pathway, promoting the release of inflammatory factors IL-6 and TNF-α, and recruiting tumor-associated macrophages [82]; the two coordinately function to establish a metastasis-promoting microenvironment: integrin mediates the adhesion of tumor cells to peritoneal mesothelial cells, while Galectin-3 binds to laminin in the peritoneal matrix, providing a microenvironment for cell colonization; Gal-1 can interact with the β1 integrin on the surface of gastric cancer cells, regulating its conformation and affinity to the ECM, thereby affecting the dynamics of cell adhesion, and playing a role in different stages of invasion and metastasis. Combined strategies include: anti-integrin αvβ6 monoclonal antibody (BG00011) and Gal-1 inhibitor [83], as well as the exploration of the combination of Galectin-3 inhibitor and anti-angiogenic drugs.

### 3.8. Ovarian Cancer

Integrin β1 and αvβ3 are key mediators of peritoneal dissemination in ovarian cancer [84]. Tumor cells bind to fibronectin and collagen in the peritoneal matrix through integrin β1, initiating colonization [85]; galectin 1 is present in high concentrations in malignant ascites, and it establishes local immune tolerance in the peritoneal cavity by inducing apoptosis of CD8+ T cells and promoting the expansion of regulatory T cells [86]. While galectin 8 is mainly nuclearly expressed in ovarian cancer, its expression level is significantly correlated with histological type, tumor grade, and stage, supporting it as an important prognostic biomarker and potential therapeutic target [87]. The synergistic mechanism lies in: Integrin signaling upregulates HIF-1α in tumor cells, thereby increasing Galectin-1 secretion. Galectin-1, in turn, enhances its affinity for extracellular matrix by binding to the glycan chain of integrin β1, promoting tumor sphere formation and chemotherapy resistance [88]. The treatment progress focuses on targeted delivery and combination immunotherapy: a triple regimen of poly(ADP-ribose) polymerase (PARP) inhibitors olaparib and niraparib with anti-angiogenic drugs bevacizumab and PD-1 inhibitors pembrolizumab shows deep remission in homologous recombination deficiency (HRD)-positive patients (Table 1).

## 4. Integrin- and Lectin-Targeted Drugs: Research Progress, Clinical Development, and Application Challenges

### 4.1. Integrin- and Lectin-Targeted Drug Research and Clinical Application Progress

#### 4.1.1. Small Molecule Antagonists

These drugs simulate or block the binding sites of integrins and ligands (ECM proteins), inhibiting their signal transduction. The classic Arg-Gly-Asp (RGD) sequence mimetics, such as cilengitide, can competitively inhibit integrins αvβ3 and αvβ5. Initially intended for anti-angiogenesis, although they did not reach the primary endpoint in the Phase III clinical trial for glioblastoma, they verified the feasibility of targeting integrins [100]. Oral small molecule antagonists targeting other integrins α4β1 and α4β7 are also under development, mainly for the treatment of inflammatory diseases and multiple sclerosis, providing a mechanism reference for application in the oncology field [101].

#### 4.1.2. Monoclonal Antibodies

Monoclonal antibodies can specifically and with high affinity bind to the specific epitopes of integrins or lectins, blocking their functions. Natalizumab is a humanized monoclonal antibody targeting integrin α4, which inhibits lymphocyte migration and has been approved for multiple sclerosis and Crohn’s disease. Its potential application in tumor immunology is being explored [102]. For lectins, such as anti-Galectin-3 and anti-Galectin-9, neutralizing antibodies are undergoing clinical trials, aiming to reverse T cell exhaustion and immunosuppression mediated by these lectins, and to be combined with PD-1/PD-L1 inhibitors [103].

#### 4.1.3. Antibody Drug Conjugates (ADCs)

ADCs link highly specific antibodies targeting integrins or lectins with potent cytotoxic drugs for precise delivery. For example, for the ADC drug SKB105 targeting integrin β6, its antibody part can recognize the highly expressed integrin β6 in various epithelial-derived tumors and, after internalization, releases the toxin monomethyl auristatin E (MMAE), selectively killing tumor cells [104]. These drugs aim to overcome the systemic toxicity of traditional chemotherapy and be effective against drug-resistant tumor cells expressing the target [105].

#### 4.1.4. Peptide Inhibitors and Peptide Analogs

Cyclic peptides or peptide analogs based on the structure of natural ligands such as RGD, Leu-Asp-Val (LDV) sequences have higher stability, affinity, and targeting. For example, ATN-161 is a pentapeptide antagonist targeting integrin α5β1, which can inhibit angiogenesis and tumor growth, and has entered clinical research [106]. These drug molecules have a small molecular weight and good tissue permeability but may have problems such as rapid metabolism and low oral bioavailability and are often optimized through formulation modification.

#### 4.1.5. Small Molecule Lectin Inhibitors

Small molecule compounds targeting the sugar recognition domain of lectins are developed to competitively inhibit their binding to sugar chains. For example, TD139 is an inhalable small molecule inhibitor of Galectin-3, originally developed for idiopathic pulmonary fibrosis, which inhibits fibroblast activation and immune regulation mediated by Galectin-3, showing a synergistic effect with immune checkpoint inhibitors in preclinical tumor models [107]. The key to the development of these drugs lies in improving their selectivity for specific lectin family members to avoid off-target effects.

#### 4.1.6. Bispecific Antibodies

BsAb can simultaneously bind to two different targets. In tumor treatment, they are often designed as “T cell connectors” or “dual signal blockers”. For example, the bispecific antibody JSKN022 targeting PD-L1 and integrin αvβ6 not only blocks the PD-1/PD-L1 immune checkpoint pathway [108] but also exerts a dual anti-tumor effect by inhibiting the signal transduction of integrin αvβ6 and the mediated endocytosis [109]. This design is expected to simultaneously overcome the immune suppression and matrix-mediated resistance mechanisms.

#### 4.1.7. Cell Therapy

By genetically engineering T cells to express chimeric antigen receptors (CARs) that can recognize tumor-associated integrins or lectin antigens, T cells are guided to specifically kill tumors. For example, CAR-T therapy targeting integrin αvβ3, which is highly expressed on the tumor vasculature and cell surfaces in osteosarcoma and melanoma, is currently being investigated in preclinical settings. The main challenge lies in the low-level expression of the target antigen in normal tissues (on-target/off-tumor toxicity) in solid tumors and the limitation of CAR-T cell infiltration and persistence in the immunosuppressive microenvironment. [110].

#### 4.1.8. Nucleic Acid Drugs

Antisense oligonucleotides (ASOs) are single-stranded oligonucleotides with sequences complementary to the mRNA of a target gene. When binding to mRNAs encoding integrins or lectins such as galectins, ASOs primarily function in two ways: by recruiting intracellular ribonuclease H (RNase H) to degrade the target mRNA, thereby preventing protein translation; or by directly blocking ribosome binding or interfering with mRNA splicing to inhibit protein expression [111,112]. For example, ASOs targeting integrin β1 effectively inhibit the adhesion and invasion of human colon cancer cells [112]. siRNA is a double-stranded RNA molecule that induces gene silencing via the RNA interference pathway. Its guide strand binds to the RNA-induced silencing complex, enabling specific recognition and cleavage of complementary integrin or lectin mRNA, leading to its degradation. For instance, siRNA targeting Galectin-1 effectively reduces its mRNA and protein levels in lung adenocarcinoma and gastric cancer models, thereby suppressing tumor cell proliferation, migration, and invasion, reversing epithelial–mesenchymal transition, and enhancing chemosensitivity [113].

Aptamers are single-stranded DNA or RNA molecules obtained through in vitro selection, capable of binding with high affinity and specificity to target proteins such as integrin αvβ3. Their advantages include low immunogenicity and ease of chemical modification. By folding into specific three-dimensional structures, aptamers precisely recognize and occupy the ligand-binding domain of integrins, competitively inhibiting their interaction with extracellular matrix proteins like vitronectin. This blocks outside-in signaling, thereby suppressing tumor cell adhesion, migration, and angiogenesis [114]. These nucleic acid therapeutics can be further integrated with delivery systems—such as nanocarriers, liposomes, or hydrogels—to achieve tumor-targeted delivery. Preclinical studies demonstrate their potential to enhance chemosensitivity, inhibit metastasis, and modulate the immune microenvironment (Table 2).

### 4.2. The Development Process and Clinical Progress of Targeted Drug Research

Research and development of integrin- and lectin-targeted therapeutics have followed a distinct evolutionary path, progressing from “single-target functional blockade” to “synergistic pathway intervention” and, more recently, toward “multifunctional precise targeting.” In the monoclonal antibody field, drugs such as natalizumab targeting integrin α4β1 and vedolizumab targeting α4β7 are primarily used in autoimmune diseases including multiple sclerosis and Crohn’s disease, confirming the safety profile of this pathway [101]. In oncology, monoclonal antibodies against integrin αvβ6 are under clinical investigation for conditions like idiopathic pulmonary fibrosis [122], while combination strategies pairing galectin-3 small-molecule inhibitors such as GB1211 with PD-L1 inhibitors have demonstrated potential to enhance anti-tumor immunity in preclinical studies [51].

Within the domain of small molecule inhibitors, peptide-based αv integrin inhibitors like cilengitide once garnered significant interest due to their oral bioavailability; however, their failure to improve overall survival in a phase III glioblastoma trial underscored the limitations of a single-target approach [123]. This outcome has subsequently driven the development of more selective agents with novel mechanisms, exemplified by the oral selective αvβ1/αvβ6 dual integrin inhibitor PLN-74809, which shows promise in clinical trials for fibrotic diseases and offers new rationale for combination therapy in related cancers [119]. Furthermore, the research frontier is increasingly shifting toward targeting multiple nodes within signaling networks—for instance, inhibiting the downstream hub FAK to create synergy with various treatments, or targeting the galectin-9/TIM-3 axis to reverse immunosuppression, representing a new exploratory direction to overcome resistance to immunotherapy [124].

### 4.3. Core Challenges in Clinical Application

The clinical translation of integrin- and lectin-targeted drugs faces a series of core challenges rooted in their complex biological mechanisms. The primary challenge arises from the significant heterogeneity of tumors and the targets themselves. Members of the integrin family, for example, display markedly different expression profiles and functions across various cancer types and even within the same tumor. While integrin αvβ3 plays a key role in breast cancer bone metastasis, integrin α3β1 may exert an anti-cancer function in prostate cancer, a functional duality that complicates the development of universal biomarkers [125]. Secondly, resistance mechanisms are complex and dynamic, involving multi-layered adaptive processes. Upon integrin blockade, tumor cells can maintain survival by activating alternative signaling pathways such as FAK/Src and PI3K/Akt [126]. Research has also revealed that proteins like OCIAD2 can interact with SNX17 to promote the recycling of integrin β1 from endosomes back to the cell membrane’s lipid raft region, thereby avoiding its degradation, stabilizing survival signals, and leading to chemoresistance [127].

Further challenges include significant off-target toxicity and the complexities of optimizing combination therapies. Given the important physiological roles of integrins and lectins in the immune system and vascular endothelium, drugs targeting them can interfere with these functions, leading to dose-limiting toxicities such as increased infection risk and impaired wound healing. Finally, systematically optimizing combination regimens with existing therapies like chemotherapy, radiotherapy, and immunotherapy presents a substantial hurdle. This includes determining the optimal dosing sequence, dose ratios, treatment duration, and how to use biomarkers to dynamically adjust these strategies—all of which require innovative clinical trial designs to address [128].

## 5. Glycan-Dependent Tumor Immune Regulation by Integrins and Lectins: Molecular Network Construction and Combined Therapeutic Strategies

### 5.1. Strategies of Integrins and Lectins in Tumor Immune Regulation and Combined Therapy

Integrins and lectins play crucial regulatory roles in the infiltration, activation, and function of various immune cells within the TME, serving as core drivers in establishing an immunosuppressive state. Regarding T cell regulation, integrin α4β1, which is highly expressed on T cells, mediates their rolling, stable adhesion, and transendothelial migration toward tumor sites by binding to vascular cell adhesion molecule-1 (VCAM-1) on vascular endothelium, making it a key molecule for immune cell homing [129]. Meanwhile, galectin-3, often highly expressed in the TME, significantly inhibits T cell function. Studies show that galectin-3 binds to glycoproteins on the T cell surface, impairs immune synapse formation, enhances TCR downregulation, and suppresses early TCR signaling, thereby raising the T cell activation threshold and weakening anti-tumor activity—a key mechanism of tumor immune escape [130]. Similarly, galectin-9 contributes to immune escape by interacting through its C-terminal carbohydrate-recognition domain with receptors such as T cell immunoglobulin and mucin domain-containing protein-3 (Tim-3) on T cells. The galectin-9-Tim-3 interaction can activate apoptotic pathways including caspase-3/7, inducing effector T cell apoptosis, particularly of Th1 and CD8^+^ T cells, which directly weakens anti-tumor immunity. This mechanism has been validated in cancers such as non-small cell lung cancer (NSCLC) and chronic lymphocytic leukemia (CLL) and is associated with poor patient prognosis. MDSCs further amplify local immunosuppression by expressing galectin-9 to induce T cell apoptosis and upregulate PD-L1 [131]. Myeloid-derived suppressor cells further amplify local immunosuppression by expressing galectin-9 to induce T-cell apoptosis and upregulate PD-L1 [132].

### 5.2. Construction of a Glycan-Dependent Immune Regulatory Molecular Network

The collaborative immune regulation mediated by integrins and lectins is highly dependent on precise molecular recognition driven by glycan modifications, forming a distinctive glycan-dependent immunoregulatory network. As carbohydrate-binding proteins, lectins exhibit strict glycan specificity in their interactions with integrins. For instance, galectin-3 preferentially recognizes the β1,6-GlcNAc-branched N-glycans on integrin αvβ3, which are synthesized by N-acetylgalactosaminyltransferase V. This binding is essential for initiating downstream pro-angiogenic signaling [133]. In contrast, sialylation catalyzed by α(2,6)-sialyltransferase 1 creates steric hindrance that negatively regulates this pathway [134]. At the core of this network lies a dynamic “integrin-lectin-glycosylation” positive feedback loop: lectins can recognize and bind to glycan structures on integrins present on immune cell surfaces, thereby altering their function [135]; conversely, upon activation, integrins can inversely regulate the expression of intracellular glycosyltransferases such as MGAT4A and MGAT5 through downstream signaling pathways like FAK/PI3K/Akt. This continuously alters the glycan modification states of immune molecules and helps stabilize the immunosuppressive microenvironment [136].

This regulatory network also deeply intersects with classical immune checkpoint pathways. Integrin αvβ3 can promote the upregulation of PD-L1 expression on tumor cells via an IFNγ-mediated STAT1 signaling pathway [137], while galectin-3 directly enhances PD-L1 expression by upregulating STAT3 phosphorylation [138]. In preclinical models, inhibiting galectin-3 significantly enhances the anti-tumor efficacy of PD-L1 blockade therapy, revealing the potential of targeting this network to improve immunotherapy outcomes [138].

## 6. Conclusions

Through precise glycan-mediated recognition and signaling coordination, integrins and lectins establish a multi-layered, dynamic regulatory network that profoundly influences tumor initiation, progression, immune evasion, and therapy resistance. This network not only directly drives the malignant behaviors of cancer cells but also systematically sustains a microenvironment conducive to tumor growth by remodeling the ECM, regulating angiogenesis, and suppressing anti-tumor immunity [4,45,139].

Although the development of drugs targeting this network has advanced, its clinical translation faces core challenges, including significant tumor and target heterogeneity, complex bypass activation mechanisms [140] and on-target/off-tumor toxicity arising from the indispensable physiological roles of integrins and lectins in normal tissues.

A dedicated discussion of this toxicity is warranted. Integrins are essential for hemostasis—platelet aggregation is absolutely dependent on αIIbβ3, whose loss-of-function causes the severe bleeding disorder Glanzmann thrombasthenia [141]—and for wound healing, where adhesion-blocking antibodies against β4 delay keratinocyte re-epithelialization and induce basement membrane splitting in human skin equivalents [142]. The concern is not hypothetical for the lectin arm either, although the risk profile differs: galectin-1, -3 and -9 function physiologically as immunosuppressive and tolerogenic regulators of immune homeostasis, so their inhibition carries a theoretical hazard of immune over-activation and impaired inflammation resolution—mechanistically opposite to the impaired immune surveillance seen with integrin blockade [143]. The clearest clinical warning remains Progressive Multifocal Leukoencephalopathy (PML): the anti-α4 integrin antibody natalizumab carries a black-box warning, with 212 confirmed cases among 99,571 treated patients (2.1 per 1000), rising to 11.1 per 1000 in patients positive for anti-JC virus antibodies with prior immunosuppressant exposure and 25–48 months of treatment [18]. Notably, however, poor tolerability has not been the limiting factor in oncology to date: in the phase III CENTRIC trial, cilengitide added no overall toxic effects, with grade 3–4 hemorrhage in only 2% of both arms, and the failure was one of efficacy [140]. The therapeutic window is therefore likely to narrow only once genuine target coverage is achieved—through higher subtype selectivity, dual targeting, or biomarker-guided enrichment—making prospective safety assessment a prerequisite rather than a retrospective concern.

Future breakthroughs will depend on a deeper understanding of the synergistic regulatory mechanisms of integrins and lectins within the TME, the development of precise dual-targeting immunotherapeutic strategies, highly specific targeted agents paired with effective biomarker combinations, and strengthened collaboration between fundamental research and clinical translation, which together are expected to substantially improve the efficacy of targeted therapies.

## 7. Future Prospects and Challenges

Although the clinical translation of the integrin-lectin collaborative network has promising prospects, it still faces profound challenges due to its complexity, dynamics, and indispensability of physiological functions, including high heterogeneity of target expression, treatment-induced bypass activation and resistance, and the narrow therapeutic window and potential toxicity caused by interfering with normal immune surveillance; in addition, there is still a lack of a biomarker system based on the characteristic spectrum of this network for precise prediction of efficacy. Given this, future breakthroughs will rely on multi-dimensional collaborative innovation: Mechanically, it is necessary to utilize technologies such as spatial multi-omics and live cell imaging to finely map the dynamic interactions of this network in different immune microenvironment subtypes in the time and space dimensions to identify the core druggable hubs; In terms of intervention strategies, the research focus should shift from traditional occupancy inhibition to more precise allosteric regulation, PROTAC-targeted degradation, or microenvironment-responsive drug delivery, in order to selectively disrupt pathological interactions and reduce off-target risks [144]. In terms of translational applications, the key lies in developing liquid biopsy or imaging biomarkers based on specific glycosylation characteristics, and designing mechanism-based sequential combination therapies accordingly- for example, first using integrin inhibitors to reshape the matrix barrier and promote immune cell infiltration, and then sequentially applying lectin inhibitors and immune checkpoint blockers to relieve T cell function inhibition [145]. Ultimately, by constructing innovative clinical trials that integrate patient-derived models and biomarker-guided approaches, the rapid transformation of basic discoveries into individualized combination treatment regimens will be promoted, thereby systematically overcoming current bottlenecks and achieving the ultimate goal of targeting this network to reverse immunosuppression and improve treatment outcomes.

## Figures and Tables

**Table 1:** The mechanism of integrins and lectins in different tumor types and the targeted treatment strategies.

Tumor Type	Integrin Role	Lectins Role	Synergistic Effects and Mechanisms	Representative Drugs	References
Liver Cancer	Integrin αvβ3: Mediates tumor cell adhesion to the ECM, activates the FAK/PI3K/Akt pathway, driving invasion, metastasis, and angiogenesis.	Galectin-3: Promotes tumor cell survival, migration, and participates in immune regulation.	Signal Enhancement: Galectin-3-binding protein (LGALS3BP) interacts with integrin αv, promoting TGF-β1 release and activation via F-actin rearrangement, synergistically driving liver fibrosis and HCC development. Interaction between integrin αvβ3 and Gal-3 enhances downstream signaling.	Cilengitide (αvβ3/αvβ5 inhibitor); Gal-3 inhibitors (e.g., GM-CT-01);	[89]
Breast Cancer	Integrin α6β1: Mediates adhesion to laminin, maintains stemness, self-renewal, and drives metastasis.	Gal-3: Secreted by tumor cells; acts as an immune checkpoint molecule inhibiting T cell function, promoting immune evasion.	Co-expression and Pathway Synergy: Integrin α6β1 and Gal-3 are often co-expressed in basal-like breast cancer stem cells, synergistically regulating stemness-related pathways like Wnt/β-catenin. Gal-3 enhances integrin β1 clustering and downstream signaling by binding to its glycans.	Drugs targeting α6 integrin; Gal-3 inhibitors (e.g., GB1211); Combined with immune checkpoint inhibitors (anti-PD-1).	[90]
Pancreatic Cancer	Integrin αvβ6: Efficiently activates TGF-β, a core molecule driving stromal fibrosis, EMT, and immunosuppression.	Gal-1: Highly expressed by cancer-associated fibroblasts (CAFs), induces T cell apoptosis, promotes fibrosis and immunosuppression.	Cascade Activation and Functional Synergy: Integrin αvβ6 releases and activates TGF-β, which upregulates Gal-1 expression in CAFs. Gal-1 activates pancreatic stellate cells (PSCs) via integrin β1, producing IL-8, enhancing NF-κB signaling, promoting tumor-stroma crosstalk and immunosuppressive microenvironment.	Anti-αvβ6 mAbs (e.g., 264RAD) combined with chemotherapy; Gal-1 inhibitors; Anti-fibrotic drugs (e.g., PEGPH20)	[91,92]
Colorectal Cancer	Integrin α5β1: Mediates binding of circulating tumor cells (CTCs) to endothelial VCAM-1, a key step in hematogenous metastasis.	Gal-3 and Gal-9: Gal-3 enhances cell adhesion and proliferation; Gal-9 acts as an immunomodulator, inducing T cell apoptosis or exhaustion.	Functional Synergy: Gal-3 enhances the affinity and stability of integrin α4β1 binding to VCAM-1, jointly promoting CTC anchoring to the vasculature. Gal-9 synergizes with integrin signaling to suppress immune responses by binding Tim-3 on T cells.	Cetuximab; Integrin inhibitors (e.g., GLPG-0187);	[93,94]
Glioblastoma	Integrin αvβ3/αvβ5: Highly expressed on tumor and endothelial cells, promoting invasion, angiogenesis, and radioresistance.	Gal-1: Involved in tumor progression, angiogenesis, and immunosuppressive microenvironment formation.	Co-localization and Signaling Crosstalk: Integrin αvβ3 and Gal-1 co-localize at the invasive front, synergistically activating Rho GTPase and other signals to drive cytoskeletal reorganization and migration; both jointly regulate pro-angiogenic factor expression.	Cilengitide; αvβ3 CAR-T cell therapy; Gal-1 inhibitors.	[95,96]
Lung Cancer	Integrin β1 and α5β1: Integrin β1 signaling is a key bypass mechanism for acquired resistance to EGFR-TKIs; α5β1 promotes cell migration.	Gal-3 and Gal-9: Gal-3 promotes metastasis and drug resistance; Gal-9 induces T cell exhaustion as a ligand for TIM-3.	Signaling Pathway Synergy: In EGFR-TKI resistance, activation of integrin β1 and upregulation of Gal-3 co-sustain downstream survival signals like PI3K/Akt, bypassing EGFR inhibition. Transmembrane protein MCAM drives resistance by activating the JAK3 signaling axis via binding to integrin β1.	Integrin β1 inhibitor combined with EGFR-TKIs; Gal-3/Gal-9 inhibitors combined with immunotherapy.	[97,98]
Gastric Cancer	Integrin αvβ6: Drives EMT and invasion by activating FAK and TGF-β signaling.	Galectin-1: Highly expressed in gastric cancer; regulates cell adhesion, EMT, and immune evasion by binding to integrins like β1 integrin.	Interaction and Functional Modulation: Gal-1 interacts with β1 integrin on gastric cancer cells, modulating its conformation and affinity for the ECM, thereby affecting the dynamics of cell adhesion and playing roles in different stages of invasion and metastasis.	Integrin αvβ6 inhibitors; Gal-1 inhibitors;	[99]

**Table 2:** Mechanisms of action and clinical applications of drugs targeting integrins and lectins.

Drug Type	Representative Drug	Target	Molecular Mechanism of Action	Development Stage/Regulatory Status	Clinical Indication (Approved vs. Investigational	Target Disease	References
Small Molecule Inhibitor	GLPG0187	Pan-αv integrins (αvβ1,3,5,6,8)	A small molecule RGD mimetic that acts as a reversible antagonist of various integrins containing the αv subunit, inhibiting cell adhesion, migration, and TGF-β activation mediated by them.	Phase Ib completed (solid tumors)	Advanced solid tumors (investigational only)	Advanced solid tumors, Bone metastasis	[115,69]
Monoclonal Antibody	Natalizumab	Integrin α4β1	A humanized IgG4 monoclonal antibody that specifically binds the integrin α4 subunit, sterically blocking its interaction with the vascular addressin VCAM-1, thereby inhibiting lymphocyte transendothelial migration.	FDA-approved (2004) for MS; later approved for Crohn’s disease	Approved: Multiple Sclerosis, Crohn’s Disease. Investigational (oncology): pulmonary metastatic osteosarcoma (Phase I/II, single-arm exploratory)	Multiple Sclerosis, Crohn’s Disease	[116]
Antibody-Drug Conjugate	SGN-B6A	Integrin β6	A humanized monoclonal antibody targeting integrin β6, conjugated via a protease-cleavable linker to the microtubule inhibitor MMAE, enabling tumor-specific internalization and drug release.	Phase I completed; Phase III ongoing (NSCLC)	Advanced solid tumors (NSCLC, pancreatic, HNSCC, etc.)—investigational	Advanced solid tumors (e.g., NSCLC, Pancreatic Cancer)	[104]
Peptide Inhibitor	A20FMDV2 and its conjugates	Integrin αvβ6	A 20-amino-acid peptide derived from foot-and-mouth disease virus, exhibiting nanomolar affinity and excellent selectivity for integrin αvβ6. It can serve as a targeting vehicle or imaging probe.	Preclinical/experimental	αvβ6-positive tumors (experimental/imaging context)	Integrin αvβ6-positive tumors	[117]
Small Molecule Inhibitor	GB1211	Galectin-3	An orally bioavailable small molecule that competitively binds the carbohydrate recognition domain of Gal-3, blocking its cross-linking with multivalent glycoconjugates and reversing T cell suppression.	Early clinical/investigational	NSCLC, HNSCC (investigational)	Non-Small Cell Lung Cancer, Head and Neck Squamous Cell Carcinoma	[118]
Small Molecule Inhibitor	PLN-74809	Integrin αvβ1/αvβ6	An oral, selective dual antagonist that occupies the ligand-binding site of these integrins, inhibiting their mechanosignaling and the activation of latent TGF-β.	Phase II (non-oncology indications)	Approved: None. Investigational: Idiopathic Pulmonary Fibrosis, Primary Sclerosing Cholangitis	Idiopathic Pulmonary Fibrosis, Primary Sclerosing Cholangitis	[119,120]
Polysaccharide Inhibitor	GM-CT-01 (Davanat)	Galectin-1, Galectin-3	Acts as a soluble competitive inhibitor for Galectin-1 and Galectin-3. By blocking the function of these immune checkpoint proteins, it remodels the tumor immune microenvironment, alleviates immunosuppression, and restores anti-tumor immune responses, demonstrating synergy with chemotherapy.	Phase I/II completed (trials terminated prematurely); no oncology approval	Approved: None (oncology). Investigational: metastatic colorectal cancer (Phase I/II, terminated for financial reasons)	Colorectal Cancer	[121]

## Data Availability

No data were used for the research described in the article.
